# Current–voltage characteristics of manganite–titanite perovskite junctions

**DOI:** 10.3762/bjnano.6.152

**Published:** 2015-07-07

**Authors:** Benedikt Ifland, Patrick Peretzki, Birte Kressdorf, Philipp Saring, Andreas Kelling, Michael Seibt, Christian Jooss

**Affiliations:** 1 Institute of Materials Physics, University of Goettingen, Friedrich-Hund-Platz 1, 37077 Goettingen, Germany; 2 4th Physical Institute, University of Goettingen, Friedrich-Hund-Platz 1, 37077 Goettingen, Germany

**Keywords:** current–voltage characteristics, perovskites, photovoltaics, polarons

## Abstract

After a general introduction into the Shockley theory of current voltage (*J*–*V*) characteristics of inorganic and organic semiconductor junctions of different bandwidth, we apply the Shockley theory-based, one diode model to a new type of perovskite junctions with polaronic charge carriers. In particular, we studied manganite–titanate p–n heterojunctions made of n-doped SrTi_1−_*_y_*Nb*_y_*O_3_, *y* = 0.002 and p-doped Pr_1−_*_x_*Ca*_x_*MnO_3_, *x* = 0.34 having a strongly correlated electron system. The diffusion length of the polaron carriers was analyzed by electron beam-induced current (EBIC) in a thin cross plane lamella of the junction. In the *J*–*V* characteristics, the polaronic nature of the charge carriers is exhibited mainly by the temperature dependence of the microscopic parameters, such as the hopping mobility of the series resistance and a colossal electro-resistance (CER) effect in the parallel resistance. We conclude that a modification of the Shockley equation incorporating voltage-dependent microscopic polaron parameters is required. Specifically, the voltage dependence of the reverse saturation current density is analyzed and interpreted as a voltage-dependent electron–polaron hole–polaron pair generation and separation at the interface.

## Introduction

At present, photovoltaic devices are mainly based on high purity elemental or compound inorganic semiconducting materials with large electronic bandwidths. The doping of such semiconductors allows for the variation in the electrical conductivity and character of the charge carriers. In this way, junctions based on p- or n-doped materials can be tailored. In these materials, the charge carriers are quasi-free, that is, the effective mass is relatively small, the mobility is large and the diffusion length of excited electron–hole pairs can be in the 100 µm range for indirect semiconductors [1].

The examination of photovoltaic materials with properties deviating from conventional solar cells can lead to new strategies for a wide variety of solar cells. In recent years, organic and other narrow bandwidth semiconductors came into the focus of research efforts [2–7]. They often result in new types of quasi-particles such as polarons (i.e., bound states of charge and lattice distortions). Polarons are present in organic semiconductors such as conjugated polymers [8] as well as some perovskite oxides [9–11]. Perovskites have the general formula *ABX*_3_, where the *A* cation in a cuboctahedral site coordinates with 12 anions, and the *B* cation in an octahedral site coordinates with 6 anions. New perovskite materials under evaluation for photovoltaic systems reveal vastly different properties ranging from narrow band gap manganite oxides perovskites with hopping transport to broad band gap lead halide perovskites [9,12–14]. For the lead halide perovskites the constituents are: *A* = CH_3_NH_3_^+^, *B* = Pb, and *X* = I, Br, Cl, mixed halides. The constituents for manganite oxide are: *A* = rare earth, alkali metal, mixed composition, *B* = Mn, and *X* = O.

The organic/inorganic halide perovskites exhibit good optical absorption and favorable electrical properties, thus offering the possibility for use in high efficiency solar cells [12–14]. Even though the junctions made of halide perovskites exhibit high open-circuit voltages, *V*_OC_ = 0.9–1.15 V [15–16], and a large carrier diffusion length, *L* > 1 μm, for the mixed halide, CH_3_NH_3_Pb_3−_*_x_*Cl*_x_* [17–18], these junctions seem not to be stable in the long term [19–20].

On the other hand, the manganite oxide perovskites are strongly correlated electron systems that exhibit a strong electron–phonon interaction. This leads to the formation of small polarons [21]. The polaron-like character of the quasi-particles in perovskite oxides provides at least two exciting issues related to photovoltaic energy conversion [22]: the possibility of light absorption by intraband excitations of charge carriers and the harvesting hot carriers due to the rather long-lived excited states [21,23]. Hence, such materials are suitable to study the pathways of photovoltaic energy conversion beyond the Shockley–Queiser limit [24] by reducing fundamental losses due to long wavelength transparency and thermalization of excess carriers generated by the short wavelength part of the solar spectrum.

For this study, junctions of p-doped Pr_1−_*_x_*Ca*_x_*MnO_3_ (PCMO) with *x* = 0.34 and n-doped SrTi_1−_*_y_*Nb*_y_*O_3_ (STNO) with *y* = 0.002 were prepared. In PCMO, the charge carriers are small polarons and doping with Ca leads to a variety of different electronic and magnetic ground states. For a certain doping range, Ca doping leads to field-induced electronic phase transitions such as the colossal magneto-resistance (CMR) and the colossal electro-resistance (CER). For the perovskite heterojunction La_0.32_Pr_0.33_Ca_0.33_MnO_3_ with 0.5 wt % Nb-doped SrTiO_3_, the influence of a magnetic field on the temperature-dependent photovoltaic effect was reported [5]. In contrast, STNO has a band gap of around *E*_g_ = 3.2 eV [25] and the reported type of charge carriers in STO varies from large to small polarons [26–27] or a mixture of both [28].

To gain more insight into the underlying mechanism of the photovoltaic effect in perovskite-based materials, it is important to analyze the properties at the interface. The electronic interface structure of conventional semiconductor p–n junctions is well-described in terms of electrochemical equilibrium of quasi-free electrons [29]. Charge carriers are transferred across the interface until a specific Fermi level of the carriers on both sides of the interface is established. Consequently, an electrostatic potential is generated, which modifies the band structure at the interface. The modified interfacial band structure is successfully described by band bending of more or less rigid electron bands. In heterojunctions, materials with different bandgaps meet at the interface. In addition to band bending, this leads to sharp discontinuities of the band structure at the interface and is modelled in the framework of a sharp junction [30].

In many perovskite oxides, the band structure is determined to a large degree by the correlation interactions [31]. Since these correlations strongly depend on the charge density and the material structure, the concept of bending rigid electronic bands at an interface can break down because of the emergence of new types of quasiparticles and order [32]. The nature of polaron quasiparticles may change during their interfacial transfer because of the variation of the electron–phonon interaction across the interface. Under a large electric field, the polaron may even dissociate as indicated by polaron simulations of polymer junctions [33]. On the other hand, the concept of the electrochemical equilibrium at the interface naturally takes into account the spatial variation of the correlation interactions and is quite successfully applied to the near-equilibrium interfacial band structure of oxide junctions [22] (see Figure 4 later in this article).

An additional important difference compared to conventional semiconductors is the small electronic bandwidth of the conduction bands in transition metal perovskite oxides. Because of the small electronic overlap between transition metal 3d and oxygen 2p states, the width of the unnormalized conduction band in the manganite Pr_1−_*_x_*Ca*_x_*MnO_3_ is of the order 1 eV [34], in contrast to Si with a bandwidth of ≈20 eV. The renormalization of the bandwidth by the electron–phonon interaction further reduces the bandwidth to a few meV [35]. This small bandwidth has strong impact on the matching of electronic states at the interface. Even after establishment of electrochemical equilibrium (which may be hindered by small charge transfer rates), the electronic overlap of narrow bands can be very small. In other words, the orbital mismatch of the electronic states on both sides of the interface may strongly affect the charge transfer process.

The width of the space charge region (SCR) at the junctions of conventional semiconductors can be well estimated in the framework of rigid band concepts, taking Debye or Thomas–Fermi screening into account. For the studied PCMO/STNO junction, the extensions of the SCR at room temperature calculated from the sharp junction model are *d*_PCMO_ = 0.2 nm and *d*_STNO_ = 10 nm, respectively [22]. Since the width of the SCR is on the order of one unit cell or even less, the rigid band model is not applied. Nevertheless, the calculated values roughly agree with the band bending region deduced from electron energy loss spectroscopy [22].

The strength of the electron lattice coupling also strongly affects the mobility of the electrons or holes. Compared to Si, where the mobility strongly depends on the doping level (µ_e_ ≈ 675 cm^2^/V·s and µ_h_ ≈ 331 cm^2^/V·s for a doping level of 10^17^ cm^−3^ [36]), the mobility in polaronic materials is several orders of magnitude smaller (≈1 cm^2^/V·s in STNO, 10^−2^ cm^2^/Vs in PCMO down to 5 × 10^−7^ cm^2^/V·s in hole-doped polymers) depending on the polaron effective mass. In addition to recombination rates, the mobility influences the diffusion length of electron–hole-type excitations. In polymer–fullerene solar cells, the diffusion length is significantly reduced down to the 10 nm range, which consequently reduces a typical device thickness [2]. For perovskite oxides, no direct measurement of the diffusion length has been reported so far.

From the experimental viewpoint, one of the main tools to study photovoltaic devices is the temperature-dependent analysis of current–voltage (*J*–*V*) curves measured across the charge separating junction. Typical diode-like characteristics are observed in the dark and under illumination. This provides a wealth of information related to the underlying microscopic processes such as excess carrier generation and recombination as well as transport properties in the bulk and across junction interfaces.

However, the insight that the *J*–*V* curves provide into microscopic processes is intimately linked to the applied analysis. It is quite remarkable that for the limiting cases of quasi-free electrons and small polarons, the analysis of *J*–*V* curves is performed in the framework of the classical Shockley theory [37]. This theory was originally developed for generation and recombination currents of quasi-free electrons. In more recent works, a Shockley-like equation describing a diode-like rectifying behavior has been derived from rate equations for generation, dissociation and recombination of polaron pairs at the interface [38–39]. Such a scenario is typical for photovoltaic energy conversion in polymer systems with small polaron charges. Hence, a more general description using Shockley's equations for different p–n junctions having a different charge carrier nature is needed. A simple equivalent circuit can be set up, where in addition to the diode, a parallel resistance, *R*_P_, and a series resistance, *R*_S_, is added. The temperature dependence of the diode parameters and the resistance contributions in the dark reflect the different underlying microscopic mechanisms and the nature of the charge carriers. *R*_P_ and *R*_S_ may reveal the typical small polaron fingerprints, the thermally activated hopping mobility [40], the nonlinear current–voltage dependence and the appearance of colossal resistance effects.

This article is organized as follows: First the key features of a Shockley-type model for homo- and hetero-junctions with large bandwidths and quasi-free electrons are introduced. Then we summarize the Shockley-type model for small bandwidth organic junctions with strongly localized charge carriers. The one diode-based equivalent circuit is then applied to the analysis of data sets collected from PCMO/STNO p–n heterojunctions. Despite the absence of a band gap above the charge ordering temperature of *T*_CO_ ≈ 240 K, photocarrier lifetimes in PCMO are of the order of ns [41]. The diffusion length of electron–hole-type excitations at room temperature is determined by EBIC. Finally, the Discussion section represents our analysis of the microscopic parameters obtained by fitting the *J*–*V* curves with and without illumination by using the Shockley-based one diode model. The previously reported presence of a band discontinuity at the interface [22] is confirmed in the dark and under illumination, exemplifying the self-consistency of the Shockley-based analysis. Furthermore, the temperature dependence of the characteristic parameters of the equivalent circuit provides insight into the transport mechanism in the junction and across the interface. The observed differences between simulated and measured *J*–*V* curves and the presence of a CER effect in the shunt resistance show the need to modify the Shockley equation with the introduction of bias-dependent microscopic parameters.

### Shockley equation for quasi-free electrons

**Figure 1:**
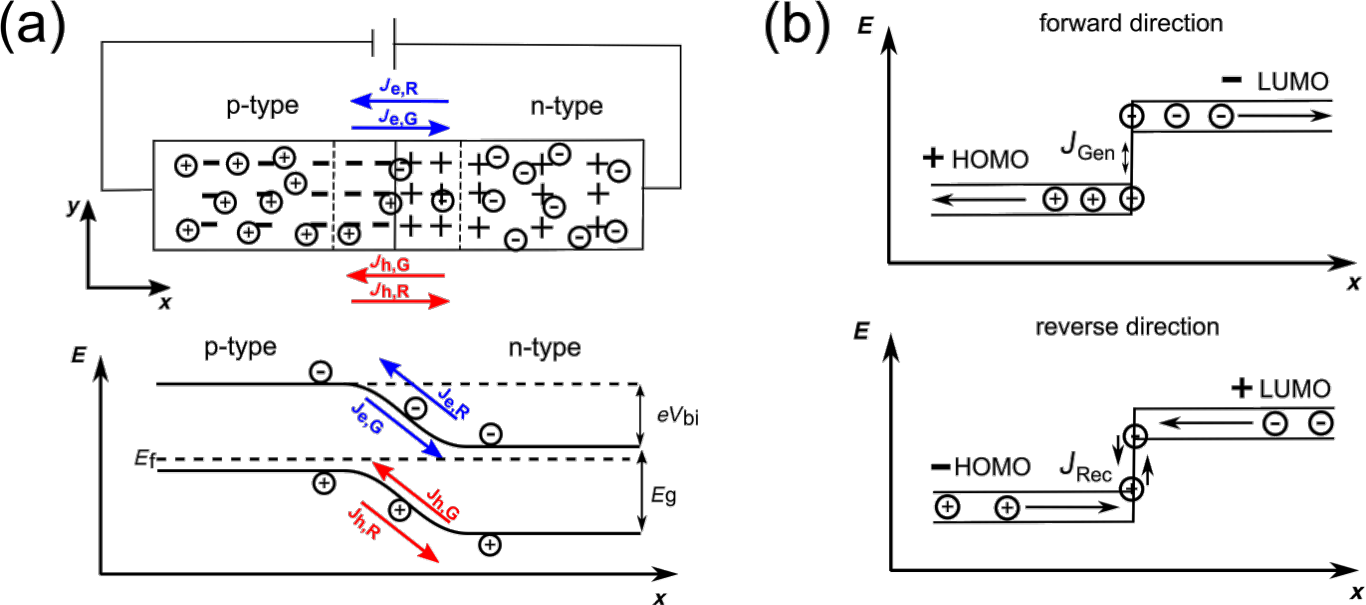
Schematic overview of electrochemical equilibrium (a) in large bandwidth inorganic semiconductors and (b) in low bandwidth organic solar cells. In both cases a recombination and generation current can be defined. In the forward direction, the recombination current is always the dominant contribution to the total current, whereas for the reverse direction, the generation current is the dominant contribution.

Let us first consider a p–n homojunction made of a semiconducting material with quasi-free charge carriers (e.g., doped silicon). Bringing n- and p-type Si in contact leads to currents that compensate for the different concentration of electrons and holes in both materials (see Figure 1a). Thus, the electrons diffuse from the n-type material to the p-type material (and holes from the p-type to the n-type material), leaving a SCR formed by immobile, ionized acceptors and donors on the p- and n-side of the junction, respectively. This current is called the recombination current density, *J*_Rec_. Thermally generated electron–hole pairs can diffuse into the space charge region where they are attracted by the electric field, resulting in electrons moving from the p- towards the n-region and holes moving in the opposite direction. This current is called the generation current density, *J*_Gen_. Thus the origin of *J*_Gen_ and *J*_Rec_ are related to the differences in the chemical and electrical potentials, respectively. In electrochemical equilibrium, there is a balance of these two currents and no net charge current, *J*_C_, flows:

[1]
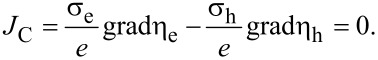


Here σ_e/h_ is the contribution of electrons and holes to the electrical conductivity, η_e/h_ is the electrochemical potential and *e* the elementary charge. As a result, a voltage drop between the n- and p-type materials, *V*_bi_, occurs. This is known as the built-in voltage or diffusion voltage.

Under bias, the electrochemical equilibrium is modified and a net charge current flows. The assumptions underlying the Shockley model of the *J*–*V* curve of a junction are [37]: (a) the voltage completely drops across the SCR; (b) a weak injection condition; and (c) no recombination occurs in the SCR.

In the ideal case, the generation current is nearly independent of the applied voltage, *V*, since the voltage has no effect on the rate of thermally generated electron–hole pairs. On the other hand, the recombination current is strongly affected by the applied voltage and is proportional to the built-in potential. For example, for the electrons from the n-region:

[2]
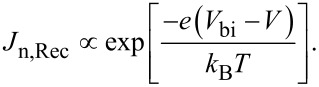


Here, *k*_B_*T* is the thermal energy. If the diode is biased in the forward direction, the barrier for the recombination current decreases and the current rises exponentially. For the reverse direction, the recombination current decreases, whereas the generation current is not influenced by the external electric field. The obtained *J*–*V* characteristic for an ideal p–n homojunction can then be described within the Shockley theory [37]:

[3]
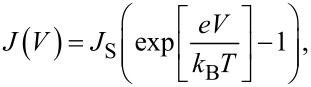


where the saturation current density, *J*_S_, can be written as

[4]
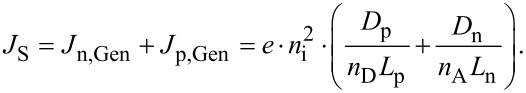


Here *D*_n,p_ is the diffusion coefficient and *L*_n,p_ is the diffusion length for electrons and holes. Far from the junction, the density of charge carriers for completely ionized donors and acceptors in the conduction or valence band is given by *n*_D_ and *n*_A_, respectively. Since the temperature dependence of the intrinsic charge carrier density, *n*_i_, is given by

[5]
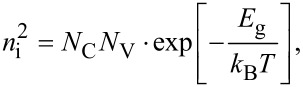


the saturation current is also temperature dependent

[6]
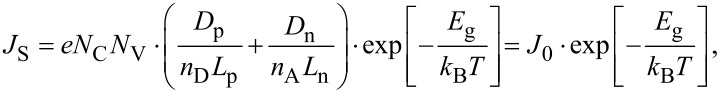


where *N*_C_ and *N*_V_ are the effective densities of states of the conduction and valence band, respectively, and *J*_0_ is nearly independent of the temperature.

### Heterojunction

If two different semiconducting materials are used, for example, a junction made of Ge and GaAs, the device is called a p–n heterostructure. One of the main differences is the presence of discontinuities in the conduction and valence bands, so-called band offsets, which can be calculated by the difference in the electron affinities, *χ*_n,p_ as

[7]
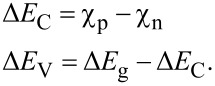


Because the materials have different band gaps and Fermi energies, the total built-in potential, *V*_bi_, is the sum of the partial built-in potentials of the semiconductor 1 and 2, named *V*_bi,1_ and *V*_bi,2_

[8]
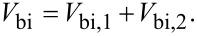


A model for the electronic structure of the interface has been developed by Anderson et al., assuming a sharp junction with band discontinuities [30]. For the derivation of the *J*–*V* curve, it is assumed that the transport mechanism is governed by injection over the barriers in the conduction and valence band. Furthermore, there are no influences of interface states taken into account that might give rise to additional space charges and barriers. If we consider a narrow band gap, p-type semiconductor 1, and a wide gap, n-type semiconductor 2, the *J*–*V* characteristics can be written as

[9]
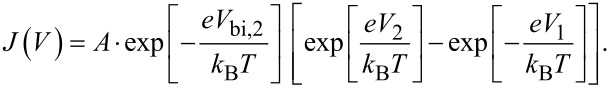


Here the partial voltage decrease over semiconductor 1 and 2 is given by *V*_1_ and *V*_2_. If we consider a p–n heterojunction, where *V*_bi,1_ > Δ*E*_C_, there is no barrier for the charge carriers in semiconductor 1 to reach the semiconductor 2 and the equation can be reformulated as

[10]
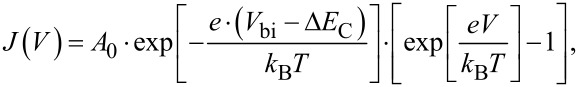


with

[11]
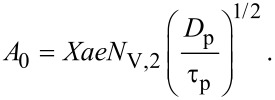


Here the assumption is made that the current is limited by the rate at which holes can diffuse in the narrow band gap material. *X* is the fraction of those carriers having sufficient energy to cross the barrier, *a* is the junction area and *N*_V_*_,_*_2_ is the effective density of states of the valence band for the semiconductor 2. This leads to a *J*–*V* curve of similar form to the ideal Shockley equation curve.

Up to this point, no other transport mechanisms such as tunneling through the interface barrier, recombination at the interface, or a voltage-dependent barrier height have been taken into account. If these processes are relevant for the *J*–*V* characteristics, the temperature-dependent *J*_S_ (the exponential prefactor in Equation 10) can be written as:

[12]
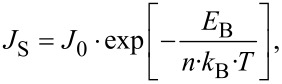


where *E*_B_ is the effective energy barrier for the transport across the interface and *n* is the ideality factor described below.

### Photovoltaic effect

Under illumination, additional charge carriers are generated and are separated in the electric field of the SCR, resulting in a photocurrent. The typical parameters characterizing the photovoltaic effect in solar cells are the short-circuit current density, *J*_SC_, and the open circuit voltage, *V*_OC_. The analysis of the temperature dependence of these parameters gives additional information about the electronic structure of the p–n interface and the transport mechanism across the interface. In inorganic junctions, the temperature dependence of the open circuit voltage is given by

[13]
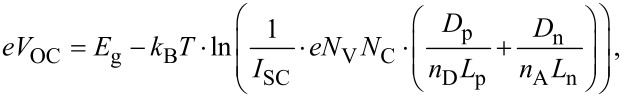


for *J*_S_ << *J*_SC_. For a heterojunction, the low temperature limit of *V*_OC_ is given by the smaller bandgap, that is, *E*_g_ is replaced by min(*E*_g1_*, E*_g2_). In the presence of a band discontinuity, the energy barrier/spike *E*_B_ which is generated at the heterointerface determines the upper limit of *V*_OC_ for *T* = 0 K [42].

### Equivalent circuit

**Figure 2:**
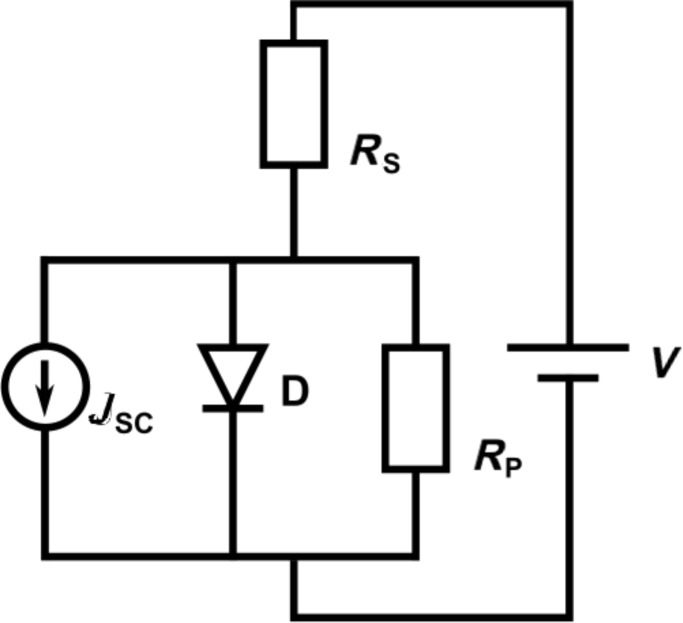
Equivalent circuit for the one diode model. The diode, D, describes the part of the circuit that represents the ideal diode equation with an ideality factor *n* = 1. *R*_S_ and *R*_P_ represent the parasitic resistances taking ohmic losses into account. *J*_SC_ represents the short circuit current density.

A real photovoltaic device is often described by an equivalent circuit. The simplest one is shown in Figure 2 and consists of one diode, which represents the ideal *J*–*V* characteristics in terms of the Shockley equation, an external power supply, a current source for the photocurrent and two ohmic resistors. These parasitic resistances describe losses, which reduce the efficiency of a solar cell. The series resistor, *R*_S_, consists of all bulk, interface and cable resistances and the parallel resistor, *R*_P_, represents losses, for example, leakage currents across the junction due to imperfections. Another important variable is the ideality factor, *n*. For an ideal diode this is *n* = 1. Evaluating the equivalent circuit yields the *J*–*V* characteristics in an implicit form,

[14]
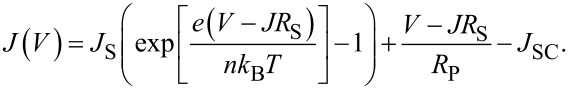


The analysis of data presented in this paper was performed using the one diode model.

### Organic solar cells: ideal diode equation for localized charge carriers, polarons

The *J*–*V* characteristics of organic solar cells formed by junctions of conjugated polymers and fullerenes are commonly described in the framework of the Shockley model [43] in combination with either one or two diode electrical circuits [44].

Since the nature of the charge carriers is fundamentally different, the applicability of a Shockley-like equation is far from obvious. In contrast to inorganic p–n junctions, where the current across the junction is due to drift diffusion and/or recombination within the SCR, the current in organic heterojunctions is carried by hole and electron-type polarons. These are formed after injection at the electrodes and can form polaron pairs at the interface.

In contrast to Si, having a large mobility, the mobility in the organic compounds is several orders of magnitude smaller. Typical values at *T* = 300 K are µ_e_ = 2.0 × 10^–3^ cm^2^/V·s in the electron-doped fullerene C61-butyric acid methyl ester (PCBM) and µ_h_ = 5.0 × 10^–7^ cm^2^/V·s in the hole-doped polymer poly(2-methoxy-5-(3′,7′-dimethyloctyloxy)-*p*-phenylenevinylene) (MDMO–PPV) [2].

Absorption of photons leads to formation of tightly bound excitons that have a very low probability of dissociation. The exciton binding energy can be high due to the low dielectric constant of the organic semiconductors and can exceed 1 eV. Charge separation is typically hindered by a high exciton binding energy, however, it can be facilitated at a heterojunction due to formation of a more loosely bound exciton–polaron pair, which can dissociate or recombine at the interface [45].

The theoretical foundation of the Shockley equation in such systems was given by Giebink et al. [38–39], who showed that for a trap-free heterojunction under stationary state conditions, the Shockley equation is

[15]
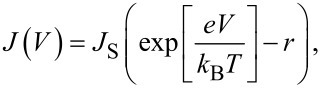


where *r* = *k*_ppd_/*k*_ppd,eq_ and *k*_ppd_ and k_ppd,eq_ denote the polaron pair dissociation rate under transport and equilibrium conditions, respectively. The polaron dissociation mainly affects the reverse direction of the junction *V* < 0, where the increased electric field at the interface facilitates the polaron dissociation and *k*_ppd_ exceeds *k*_ppd,eq_. This can typically be observed in organic junctions as an increasing reverse saturation current with increasing reverse bias. More generally, *r* > 1 can evolve due to any bias dependence of the generation current. Such an effect is disregarded in the Shockley model. However, in the forward direction, *k*_ppd_ approaches *k*_ppd,eq_ and Equation 15 reduces to the conventional Shockley equation with ideality factor *n* = 1.

Disorder and polycrystalline structure have a strong impact on the electrical transport in organic junctions, since the polarons can be trapped at defects. Consequently, the absolute value and the temperature dependence of *R*_S_ strongly depend on disorder. Since either the electron- or the hole-type polaron can be trapped, the resulting two different bimolecular recombination processes can be modelled as two different currents, which thus gives rise to an effective two diode Shockley equation with two reverse saturation currents and two ideality factors [38–39]. The contributions of both currents depend on the balance of the voltage drop across the junction as well as their characteristic trap temperatures.

The origin of the open circuit voltage, *V*_OC_, has been controversially discussed for many years. Indeed, it shows a linear increase with decreasing temperature [43,46]. Currently, there seems to be an agreement that the energy difference between the HOMO of the donor and the LUMO of the acceptor modified by the polaron binding energy controls the low temperature limit [47]. The resulting open circuit voltage is described by [38–39]

[16]
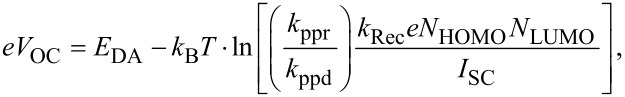


where *E*_DA_ is the energy difference between the HOMO of the donor and the LUMO of the acceptor modified by the polaron binding energy. The short circuit current density, *J*_SC_, increases with increasing temperature, reflecting the thermally activated hopping conductivity of small polarons. It should be noted, however, that this trend can be overlaid by temperature dependent changes the in morphology of the active layer [44].

The dominating loss mechanisms of organic solar cells are still under debate [48]. There seems to be some evidence that genuine (intramolecular) recombination can be disregarded compared to bimolecular recombination. The latter is due to recombination of mobile electrons and holes at the interface. The question of whether bimolecular recombination is typically affected by localized states in the band gap (similar to Shockley–Read–Hall recombination at deep traps in inorganic semiconductors) or if it involves the recombination of free carriers is highly debated (see, e.g., [45]).

Kirchartz et al. [49] introduced a voltage-dependent ideality factor (for both with and without illumination), in order to study recombination mechanisms in polymer–fullerene solar cells. They concluded that in their devices, the recombination is a trap-assisted recombination at lower voltages and surface recombination at higher voltages. However, intramolecular recombination of excitons at traps within single-blend compounds depends strongly on the exciton binding energy. Theoretical estimates show that increasing the binding energy, *E*_B_, from values of ≈*k*_B_*T* to 0.2 eV will increase the recombination rate by two orders of magnitude [2]. Typically, an experimental value for *E*_B_ in polymer solar cells is in the range of 0.2–0.4 eV.

### Modeling diffusion length determination by EBIC

The charge carrier diffusion length, *L*, is an important parameter to determine the recombination-limited charge transport processes in electronic devices [50]. It is connected to the charge carrier lifetime, τ, and mobility, μ, by the Einstein relation:

[17]
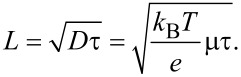


While the lifetime is determined by recombination and relaxation processes, the mobility is inherent to the material system. From the simple Einstein relation, the diffusion coefficient is proportional to the mobility. This leads to typical diffusion lengths in the µm and nm range for inorganic and organic semiconductors, respectively. This large difference is due to the fact that the typical mobility in organic and inorganic semiconductors differs by several orders of magnitude. Given that the mobility in the PCMO–STNO system is larger than that of organic semiconductors, a diffusion length on the order of that of organic semiconductors is expected. However, the situation may be different for excited charge carriers in a correlated material system as the applicability of the simple Einstein relation is questionable: Here, the assumption of a non-degenerate system in thermal equilibrium may not hold (see, e.g., [51]). As a consequence, it is necessary to directly determine the diffusion length in a PCMO–STNO p–n junction.

An established technique to measure the charge carrier diffusion length in devices with p–n junctions is by mapping the electron beam-induced current (EBIC) across the sample without any applied voltage (see, e.g., the review by Leamy [52]). Injected high-energy electrons excite electron–hole pairs, which are subject to diffusion in the sample. In the limit of weak injection, diffusion is limited by minority charge carriers. A typical experimental method for measuring the minority charge carrier diffusion length, *L*, is to vary the beam acceleration voltage, moving the excitation maximum perpendicular to the p–n interface, or by preparing a wedge-shaped layer to vary the depth of the interface in the sample. For a point-like source generating electron–hole pairs with a rate, *G*, at a distance, *W*, from the p–n interface, the resulting EBIC current is [50]

[18]
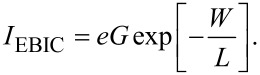


If *W* and *L* are of similar size, varying *W* allows the *L* of the minority charge carriers to be determined.

A more realistic diffusion model for charge carriers in solids incorporates the extension of the generation volume produced by a penetrating electron beam. This is especially important if the extension of the generation volume is of the order of the diffusion length. As the penetrating electrons suffer multiple scattering events with ions and electrons constituting the solid, they gradually lose their initial energy, frequently resulting in a pear-shaped generation volume [53]. This shape mainly depends on the initial electron energy, which is determined by the beam acceleration voltage and the density of the solid. It can be described by an analytical function [53] or simulated by a Monte Carlo method [54].

In this work, we measure an EBIC linescan from a p-doped to an n-doped region as a cross-section in order to extract the diffusion length. This eliminates the influence of the sample surface structure and layer thickness in addition to reducing the generation volume. In order to take the generation volume into account, the measured linescan must be compared to a simulation. For this, we convolute a simulated generation volume with a function describing the fraction of generated charge carriers contributing to the EBIC signal. For the case without diffusion processes, this function is given as

[19]
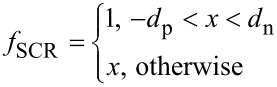


where *d*_p_ and *d*_n_ are the width of the space charge region on the p- (negative *x*) and n-side (positive *x*), respectively. This is equivalent to the assumption that all charge carriers generated in the range of the strong electric field within the space charge region are charge separated and contribute to the EBIC signal. For the case of the diffusion lengths *L*_p_ and *L*_n_, excited charge carriers in a certain area around the space charge region will also contribute to the EBIC signal. Thus, the distance from the space charge region can be exponentially weighted:

[20]
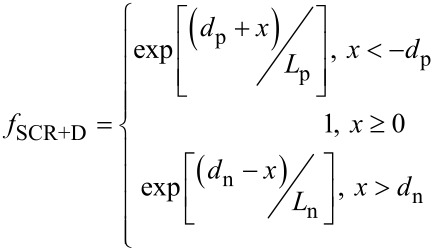


Assuming a uniform generation function in both regions, and using the convolution functions *f*_SCR_ and *f*_SCR+D_, an integrated EBIC linescan, Σ*I*_EBIC_, can be described for both cases. By dividing these two factors, the integrated generation volume is canceled out and leaves only:

[21]
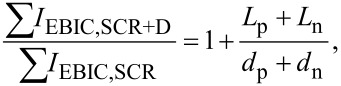


which can be used as a robust estimate for *L*_p_ + *L*_n_ if the width of the SCR is known.

## Results

### EBIC measurements

**Figure 3:**
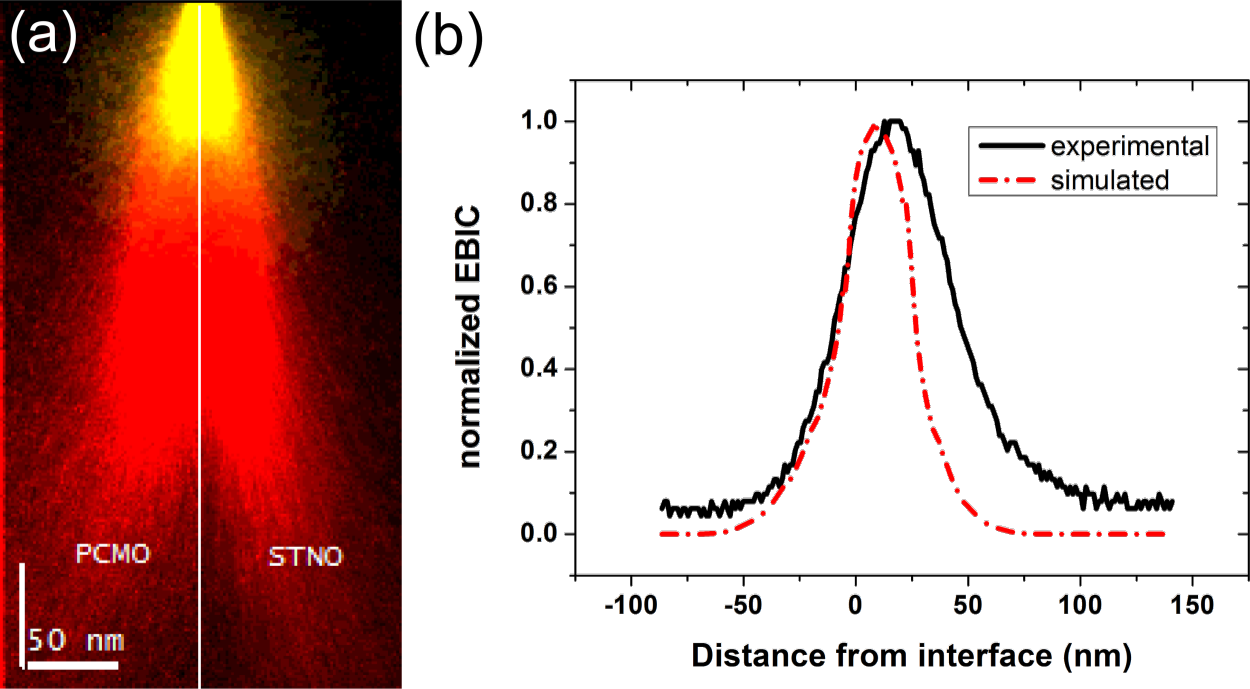
(a) Cross section of a simulated electron beam generation volume directly at the PCMO–STNO interface for electron beam acceleration voltages of 2 kV (yellow, bright) and 10 kV (red, dark). (b) Measured EBIC signal for a 2 kV line scan across the PCMO–STNO interface and corresponding simulation (see text), normalized to their maximum.

The measurements were performed at an electron beam acceleration voltage of 2 kV, as the generation volume is smallest there (see Figure 3a). Thus. it represents the situation closest to the ideal case of a point-like electron–hole pair generation source. Using even smaller acceleration voltages did not result in measureable EBIC in our setup. A cross-section lamella of the sample was prepared by means of a focused ion beam microscope. An EBIC scan across the p–n interface is shown in Figure 3b, together with a simulated EBIC linescan, taking into account only the generation volume and the space charge region.

The electron beam generation volume was calculated using the CASINO implementation of a Monte Carlo simulation developed by Drouin et al. [55]. In the simulation, we use the SCR width *d*_PCMO_ = 0.2–2.5 nm (Figure 3b for *d*_PCMO_ = 2.5 nm) and *d*_STNO_ = 27 nm, as suggested by Saucke et al. for junctions of the same materials [22]. As illustrated in Figure 3a, the differences in the generation volume on both sides of the junction are negligible. Therefore, we assume the same generation volume for PCMO and STNO.

Comparing the two linescans clearly shows the experimental curve to be broader than the simulated one, which manifests the contributions of excess carriers generated outside the space charge region (i.e., the finite diffusion lengths in STNO and PCMO). We then integrated both linescans and applied Equation 21 using the simulated, *I*_EBIC−S_, and the experimental, *I*_EBIC,S+L_. This leads to a combined diffusion length, *L*_PCMO_* + L*_STNO_ = 21.4(2) nm. The noticeable asymmetry in the experimental EBIC linescan indicates that a larger part of the combined diffusion length can be attributed to *L*_STNO_.

### Current–voltage characteristics without illumination

**Figure 4:**
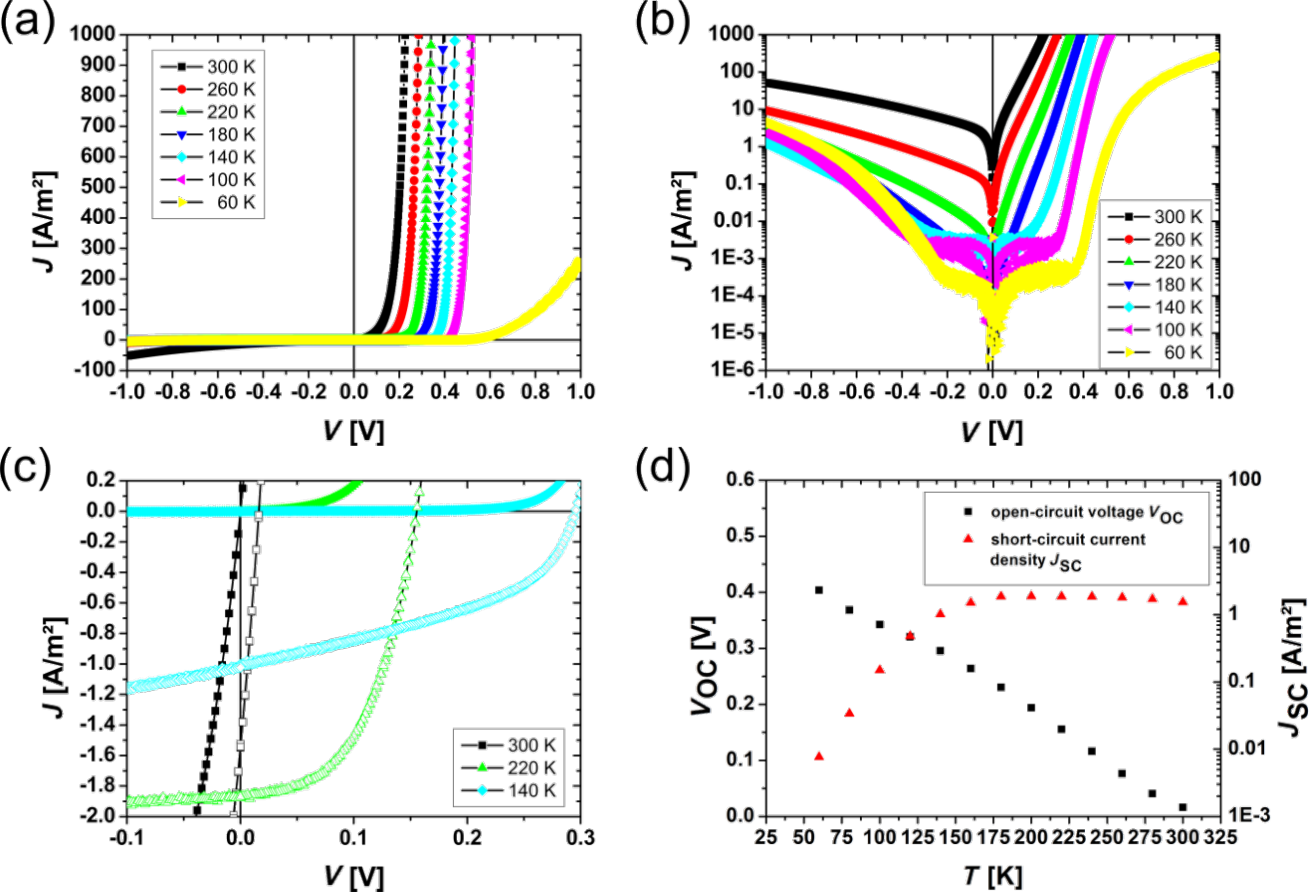
Temperature dependence of the *J*–*V* characteristics for the PCMO–STNO junction: (a) in a linear and (b) in a semi-logarithmic illustration. Over the whole measured temperature range the *J*–*V* curves show rectifying behavior. (c) Comparison of *J*–*V* curves with and without illumination. (d) The open circuit voltage increases for decreasing temperature and the short circuit current breaks down at a temperature around 180 K.

The measured current–voltage characteristics of the analyzed manganite–titanate junction are summarized in Figure 4, where *J* is the current density. For all measurements at different temperatures, the rectifying characteristic of the junction can be recognized. By decreasing the temperature, a plateau evolves for the reverse direction as well as for the forward direction for |*V*| ≤ 0.2 V. Furthermore, the exponential increase of the current is shifted to higher voltages in the forward direction.

In Figure 4c the *J*–*V* curves with and without illumination are compared. A clear photovoltaic effect is visible for all measured temperatures. Even at 300 K, without a band gap in the PCMO, the photovoltaic effect is visible. The open circuit voltage, *V*_OC_, increases with decreasing temperature, while the short circuit current density, *J*_SC_, is constant until the temperature reaches values below *T* = 140 K. Below this temperature, *J*_SC_ breaks down and decreases exponentially (see Figure 4d). In this work, the collected data sets are analyzed within the one diode model by two methods. Only the forward branch of the *J*–*V* curve is used to determine the four parameters, *J*_S_, *n*, *R*_S_ and *R*_P_ and the resistance is treated as ohmic.

**Figure 5:**
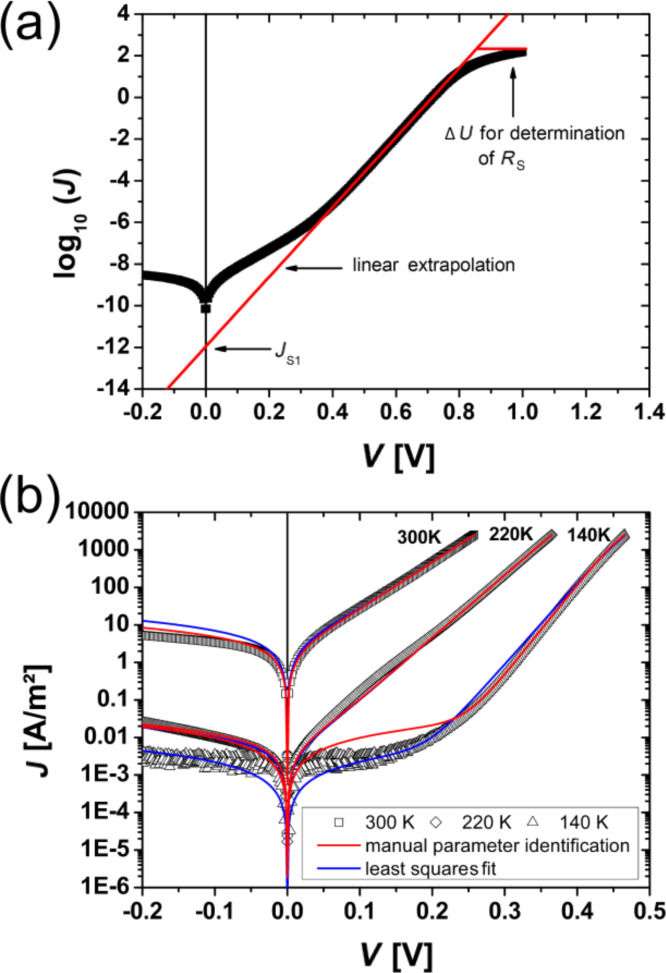
(a) Illustration of the manual parameter identification method, (b) Comparison of the measured data and the two different analysis methods for 300 K, 220 K and 140 K (open black symbols). The red line shows the result of the manual parameter identification, while the blue line is the result of the least squares fit.

**(i) Manual parameter identification:** Here the equivalent circuit is used, which is described in Figure 2, and the analysis is illustrated in Figure 5a. The assumption is made that the influence of the four parameters becomes dominant in different regimes in the forward direction. For small values of the voltage, the voltage mainly drops over the parallel resistance, *R*_P_. The parallel resistance can be determined by fitting the *J*–*V* curve linearly in a small region around *V* = 0. Since the influence of *R*_S_ is neglected, this value describes the lower limit of *R*_P_. In the intermediate voltage range, the current is governed by the influence of the diode and thus the ideality factor as well as the saturation current density can be extracted in this region. Therefore, the *J*–*V* curves are plotted semi-logarithmically and fitted linearly at the point with highest local slope in the forward branch. The ideality factor can be calculated from the slope and the saturation current is given by the ordinate intercept. For high values of the applied voltage, the current is limited by the series resistance, *R*_S_. To determine the series resistance, the difference between the linear extrapolated curve from the linear fit and the measured curve is calculated by *R*_S_ = Δ*V*/*I*_max_.

**(ii) Parameter identification with least squares fit:** The second way to analyze the *J*–*V* curves is by performing a least squares fit of the implicit Equation 14 for *J*_SC_ = 0 using a fitting routine. Here the trust region, reflective algorithm implemented in the program MATLAB was used. The four unknown parameters were limited to physically conceivable lower and upper limits. To find the best result within these bounds, the fit was performed by using uniformly distributed starting points within the bounds and the best parameter set was evaluated. Since the slope of the *J*–*V* curve is very different for low and high voltages, the sensitivity of the fit routine was adjusted accordingly.

Figure 5b shows typical results for both analysis methods for measurement temperatures of 300 K, 220 K and 140 K. Both methods correctly reconstruct the measured *J*–*V* curve in the forward direction for 300 K and 220 K. At a measurement temperature of 140 K, the manual parameter identification only fits to the linear part of the *J*–*V* curve, where the influence of the diode is dominant and overestimates the current in the low voltage regime. In contrast to this, results from the least squares fit are in good agreement with the whole forward branch. Both methods do not include any breakdown mechanism in the reverse direction. Thus, the reverse direction cannot be described well in the framework of the one diode model without any modification.

**Figure 6:**
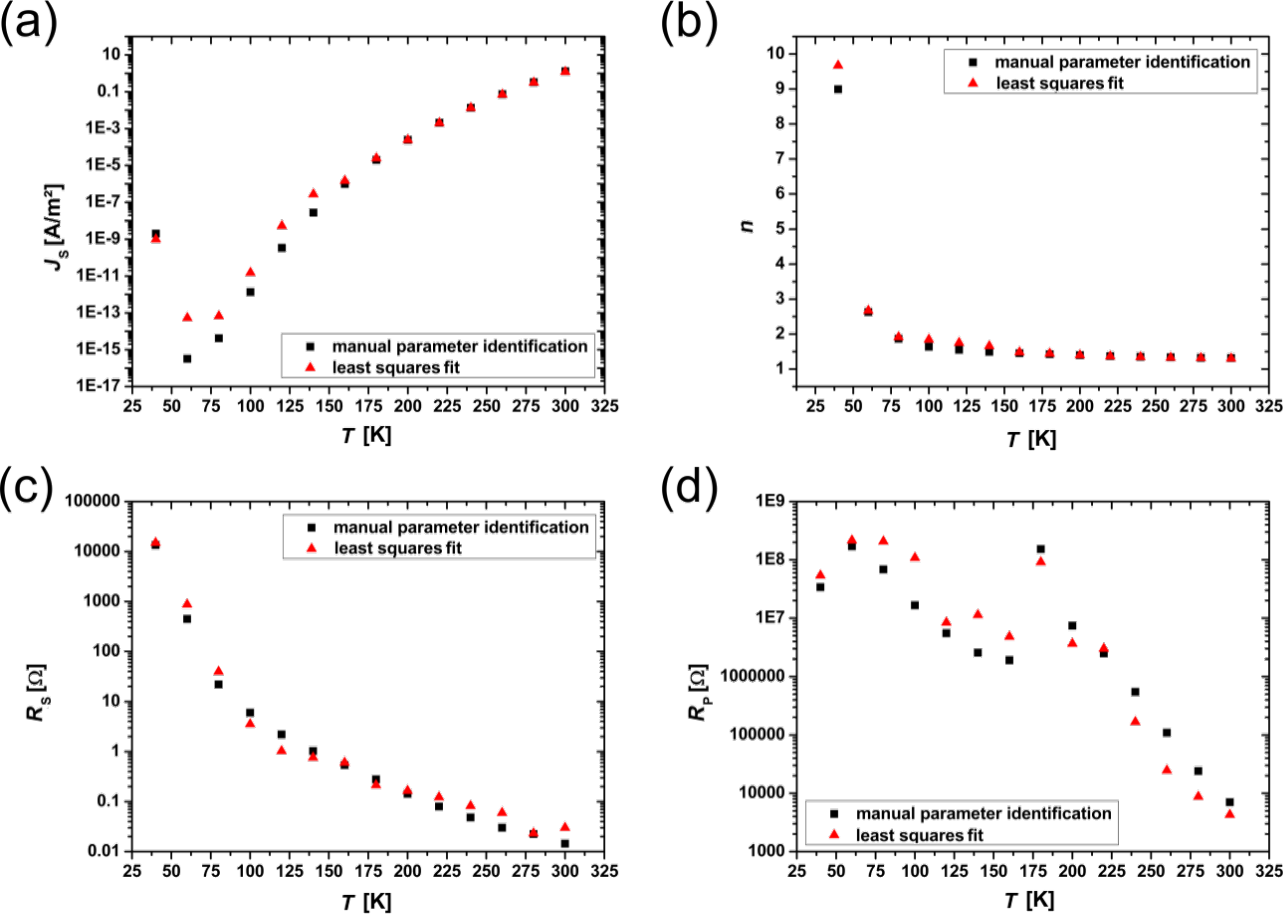
Overview of the temperature dependence of the extracted diode parameters for the two analysis methods. The black squares show the manual parameter identification results and the red triangles show the least squares fit results. (a) Saturation current, *J*_S_, (b) ideality factor, *n*, (c) series resistance, *R*_S_, (d) parallel resistance, *R*_P_. While *J*_S_ decreases with decreasing temperature, *n* as well as *R*_S_ increases. *R*_S_ shows the characteristic behavior of thermally activated transport and seems to be dominated by the PCMO resistance. *R*_P_ increases with decreasing temperature and shows two different regimes of exponential increase with a different slope.

The results for *J*_S_, *n*, *R*_S_ and *R*_P_ are plotted in Figure 6 for both methods. With decreasing temperature, the saturation current, *J*_S_, decreases exponentially over several orders of magnitude. This is in good agreement with the theoretically predicted temperature dependence (see Equation 12). For a wide temperature range, the ideality factor, *n*, increases slowly for values below 2 and strongly rises above *n* = 2 below *T* ≈ 80 K. This may indicate tunneling enhanced recombination at the interface or in the SCR [56]. The resistance, *R*_S_, shows the typical temperature dependence of a thermally activated transport process, where the resistance increases for lower temperatures and therefore is mainly dominated by the PCMO bulk resistance. In contrast to this, the parallel resistance shows two different regimes of exponential increase with a different slope in the semi-logarithmic plot. This is similar to the resistance drop caused by the CER effect in PCMO [9].

## Discussion

In the following, we discuss the temperature dependence of the determined parameters of the one diode model, in order to gain insight into the interfacial charge transfer and separation processes of polarons. The polaronic nature of the charge carriers is visible in the thermally activated hopping transport in the series and the CER-like resistance drop in the parallel resistance. Furthermore, the voltage dependence of the saturation current is discussed. We compare our results to those obtained from thin film electric transport measurements in lateral geometry from the literature. For the analysis, we use the parameters determined by the method of manual parameter identification. While the fit was found to describe the whole *J*–*V* curve in the framework of the one diode model, the manual method is more sensitive to the evaluation of the different parameters in a certain region of the *J*–*V* curve, and is thus expected to lead to more accurate parameters.

### Applicability of the Shockley-based one diode model

The temperature dependence of the reverse saturation current is given by Equation 12 and its energy barrier can be determined by

[22]
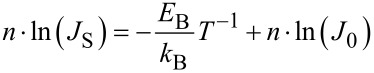


**Figure 7:**
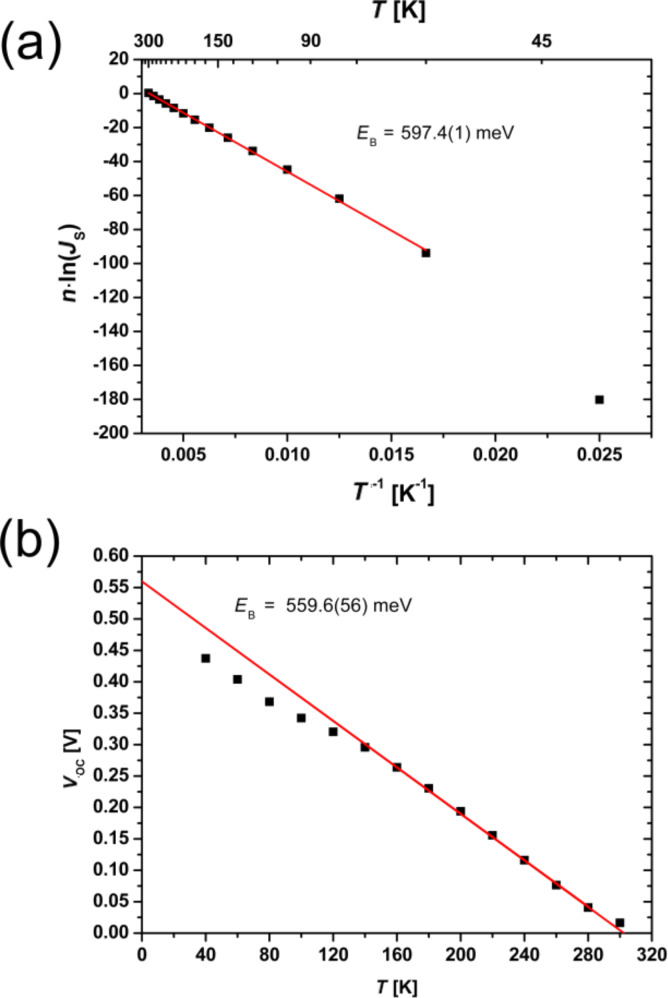
Determination of the energy barrier *E*_B_: (a) from the diode parameter analysis of the *J*–*V* characteristics without illumination *E*_B_ = 597.4(1) meV, (b) from the linear extrapolation (towards *T* = 0 K) of the open circuit voltage *E*_B_ = 559.6(56) meV. The calculated energy barrier determined by the *J*–*V* curves with and without illumination has nearly the same value.

In Figure 7a the product of *n*·ln(*J*_S_) is plotted versus the inverse temperature. From this, an energy barrier of *E*_B_ = 597.4(1) meV was calculated. According to Saucke et al. [22] this energy barrier is interpreted in the model for a heterojunction as evidence for the presence of an energy spike (band offset) in the conduction band. The theoretical value of the band offset can be approximately calculated by the difference of the work functions of the p- and n-doped material. For the materials used, these are *W* = 4.9 eV for PCMO [57] and *W* = 4.13 eV for STNO [58]. Therefore, the expected barrier height is Δ*W* = 770 meV. The slightly smaller value of the experimentally determined *E*_B_ compared to Δ*W* can be explained by a slight interdiffusion of *B*-cations at the p–n hetero-interface on the order of less than 1 nm [22], which may induce new states.

The energy barrier can also be determined from *J*–*V* curves under illumination if the temperature dependence of the open circuit voltage is taken into account, as shown in Equation 13 and Equation 16. Both equations are linear in temperature and the slope is mainly influenced by the properties of the materials. For example, one parameter in Equation 16 is the dissociation rate of exciton polaron pairs. In this work, we focus only on the intercepts of Equation 13 or Equation 16, which represent an energy barrier in both cases. By fitting in the linear region of *V*_OC_ and extrapolating towards *T* = 0 K (see Figure 7b), we obtain an energy barrier of *E*_B_ = 559.6(56) meV, which can be interpreted as the same barrier calculated from *J*–*V* curves in the dark. The result that the same value for the energy barrier is obtained from analysis of transport properties in the dark and under illumination (Equation 22 and Equation 16, respectively) confirms the consistency of the analysis and applicability of the Shockley-based model as a first step.

Conventionally, in large bandwidth inorganic semiconductors, an ideality factor with a value between 1 < *n* < 2 is seen as evidence for a contribution of Shockley–Read–Hall recombination. Therefore, in many cases, an improved fit of the *J*–*V* curves of p–n junctions can be obtained by using a second additional diode with an ideality factor of *n* = 2. If the ideality factor reaches values of *n* > 2, this could be an indication of tunneling enhanced recombination at the interface or in the SCR occurs [56]. Since these models and parameters are derived for quasi-free electrons, they cannot be easily transferred to solar cells made of oxides with strongly correlated electrons.

Another reason to introduce a second diode is given by Giebink et al. [38–39]. They introduce a second diode in organic systems in order to take the voltage dependence of different charge carrier recombination mechanisms into account. Here, hole or electron-type polarons can change their character from trapped to mobile, respectively. In our model system, the last effect is not taken into account, since the mobile carriers in the STNO are always electrons for all voltage ranges.

Even if values for the ideality factor are between 1 < *n* < 2 for higher temperatures, for our results, it is reasonable to consider only one diode because the dominating part of the current originates from the SCR. At low temperatures, the ideality factor is *n* > 2, which suggests the transition from thermionic emission to tunneling across the interface [56]. Indeed, due to the lower thermal population of phonon states, small polaron mobility at low temperatures generally exhibits an increasing tunneling fingerprint [59]. In order to improve the fit of the *J*–*V* curves for polaronic systems, it seems to be more reasonable to take the voltage dependence of the microscopic parameters into account, such as a parallel resistance, *R*_P_(*V*), rather than introducing a second diode.

### Thermally activated transport of small polarons and correlation effects

**Figure 8:**
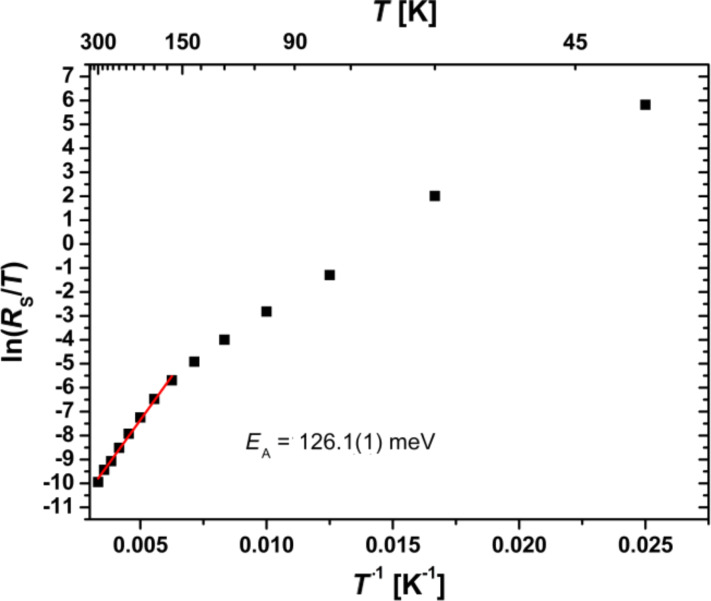
Determination of the activation energy, *E*_A,RS_, for thermally activated hopping transport from the series resistance, *R*_S_. At lower temperatures the resistance is below the values expected in the thermally activated hopping theory. Here the probability of polarons tunneling to neighboring sites is enhanced.

In Figure 8 the Arrhenius plot of *R*_S_/*T* is shown. At temperatures below half of the Debye temperature, Θ_D_/2 ≈ 160 K, the probability of polaron tunneling between neighboring sites increases. Therefore, the measured resistance is below the expected value in the model of thermally activated hopping in that case [40]. Consequently, we only use the slope from the temperature range *T* > Θ_D_/2 to determine the activation energy of the thermally activated hopping of small polarons. According to Bogomolov et al. [40], in the adiabatic limit, the resistivity can be written as

[23]
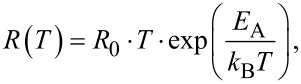


with a prefactor, *R*_0_. For the measured *J*–*V* curves, the activation energy is calculated to be *E*_A,RS_ = 126.1(1) meV. This value is in agreement with results found for PCMO in the literature, *E*_A,Lit_ = 132 meV [9]. Thus, the bulk resistance of the PCMO seems to be the dominating contribution to the series resistance, *R*_S_. The experimental value is slightly smaller because of the large electric field on the order of *E* = 10^7^ V/m in a cross-plane measurement geometry. This value considerably exceeds that in lateral measurement geometries. A strong electric field leads to a reduction of the activation energy, *E*_A_ [9].

**Figure 9:**
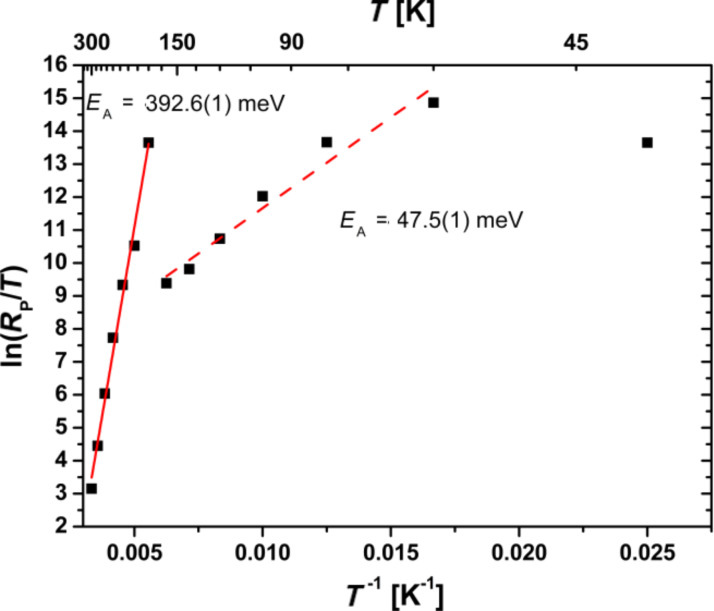
Determination of the activation energy, *E*_A,RP_, of the thermally activated hopping transport for the parallel resistance, *R*_P_. At high temperatures, the activation energy is on the order of the polaron formation energy. The second regime can be modified by the influence of a strong electric field and can be seen as an effective *E*_A,RP_*.*

In Figure 9 the Arrhenius plot of *R*_P_/*T* is shown. For high temperatures, the obtained activation energy of *E*_A,RP_ = 392.6(1) meV for *R*_P_ is on the order of the polaron formation energy in PCMO. The presence of two branches in the Arrhenius plot in Figure 9, hints at the influence of the colossal electro-resistance (CER) [9,60], which is caused by correlation effects of polarons in manganite oxides. The reduced *E*_A_,_RP_ at lower temperatures is due to an electric field-induced transition to driven polaron states and a related reduction of the activation energy for polaron transport [9]. A similar effect has been observed in PCMO–STNO junctions for the series resistance, *R*_S_ [22]. In addition, current-induced melting of charge-ordered domains is observed [61]. Since the CER in bulk PCMO samples is visible in the temperature range, where the charge ordered and disordered phase coexist and the formation of percolation paths depends on the structure as well as on the electrical pre-history, the determination of a critical electrical field is hardly possible. We assume that in our devices, the observed CER in *R*_P_(*T*) stems from such polaronic processes at the interface. This is additionally evidenced by the observation of an interfacial colossal magneto-resistance (CMR) effect at metal–PCMO interfaces [62]. In contrast to the observation of a CER in *R*_S_(*T*) by Saucke et al. [22], in the junctions studied here, the dominating contribution of *R*_P_ could originate from a higher interface resistance.

### Bias dependence of the reverse saturation current

**Figure 10:**
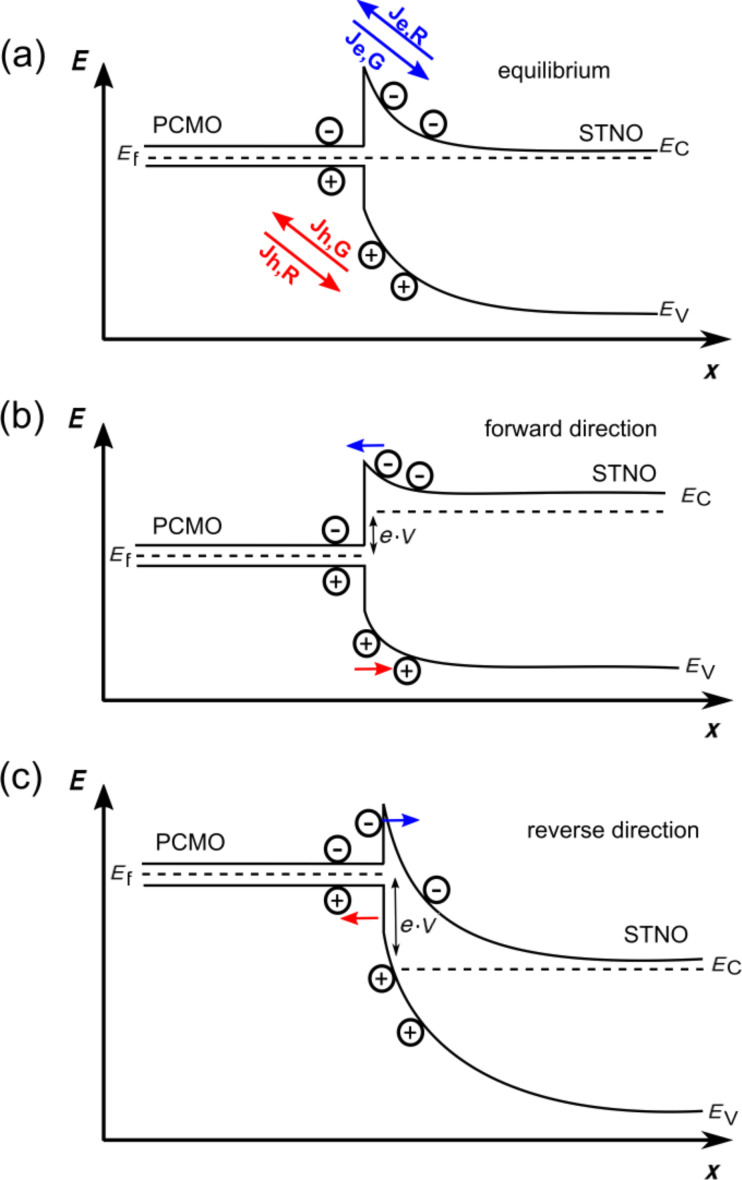
Schematic band structure of a PCMO–STNO heterojunction (a) at zero bias, (b) in the forward direction and (c) in the reverse direction.

A strong voltage dependence effect is visible in the reverse saturation current. This can be discussed according to Figure 10, where the expected simplified band diagram of the manganite–titanate junction is shown for electro-chemical equilibrium and with applied voltage in both forward and reverse directions. We disregard here all changes of the electronic structure of the PCMO near the interface due to local variations of the correlation interactions and the small screening length and assume that the main voltage drop happens in the STNO. If the diode is biased in the forward direction, the barrier for the recombination current is decreased, whereas it is increased for the reverse direction. Therefore, the generation current should be the dominating contribution to the total current measured in the reverse direction. The energy barrier calculated from the Arrhenius plot of *n·*ln(*J*_S_) has been interpreted as the presence of a band offset in the conduction band. For this reason, the transport in the reverse direction of the solar cells is governed by thermally generated charge carriers in the PCMO, which must overcome the barrier to diffuse into the STNO. If the applied voltage in the reverse direction is high enough, the barrier could be reduced or become narrower. In this way, thermally activated as well as tunneling induced charge transfer is facilitated, leading to an increasing saturation current.

Since the results from the least squares fit method describe the influence of the saturation current on the whole *J*–*V* characteristic more accurately, it is used to compare the reverse saturation current calculated on the basis of the one diode model with the measured *J*–*V* curves.

**Figure 11:**
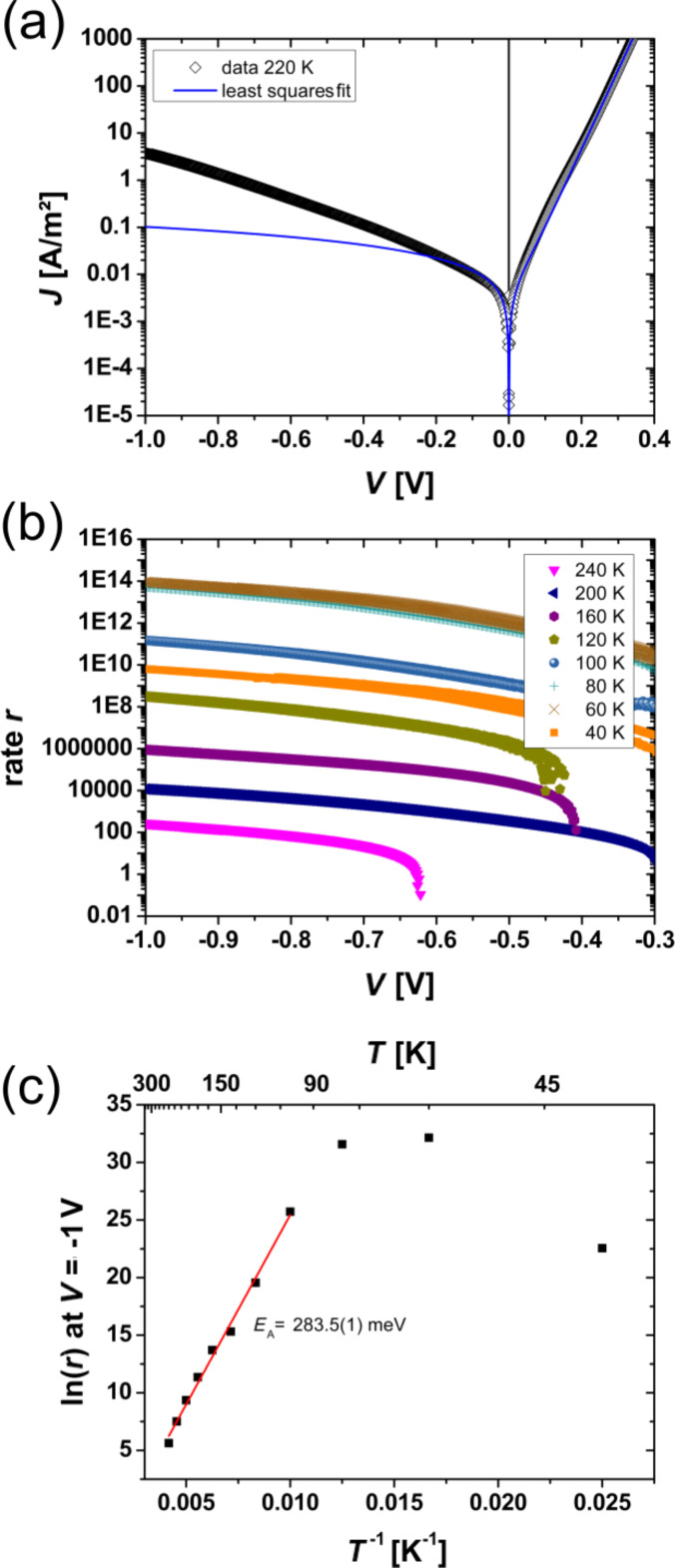
(a) Comparison of *J*–*V* curves from the least squares fit and exemplarily data at a temperature of 220 K, (b) extracted generation rate *r* as a function of the applied voltage for temperatures 40 K < *T* < 240 K, (c) determination of the energy barrier, *E*_B_, for an applied voltage of *V* = −1 V.

In Figure 11a this comparison is shown for a temperature of 220 K. For the reverse direction, a significant difference between the expected *J* (based on the determined *R*_P_) and the measured *J* is observed. Therefore, an additional voltage dependence of the reverse saturation current exists that cannot be described in the framework of the Shockley model. The differences between the measured curve and the least squares fit data can be expressed as the rate, *r*, in Equation 15 given by

[24]
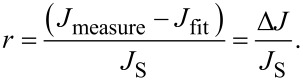


In Figure 11b the rate, *r*, is plotted against the voltage, *V*. At a temperature below 240 K and a voltage |*V|* > 0.7 V in the reverse direction, the rate is clearly higher than one. For higher temperatures or smaller voltages, this effect is too small. The presence of a finite *R*_P_ clearly cannot describe the full bias dependence of *J* in the reverse direction, since it determines only the small voltage regime of the solid curve given by the one diode model. In the forward branch of the curve for high voltages, the series resistance *R*_S_ limits the current density and a voltage-dependent *R*_P_ cannot be extracted. Thus, the influence of a voltage-dependent parallel resistance should be only visible in the large voltage regime of the reverse saturation current. Since the forward direction of the *J*–*V* curve is not strongly affected by the voltage dependence of *R*_P_, we assume that the dominant contribution to the current in the reverse direction stems from a voltage dependence of *J*_S_, where breakdown mechanisms can be excluded. The measurements are performed during the cooling down and heating of the solar cell and deliver reproducible data (the data from heating process is not shown here). In contrast to this observation, an electrical breakdown would irreversibly change the junction. In comparison to the process introduced by Giebink et al. [38–39], here, the rate *r* may not represent a pure polaron pair dissociation rate. This is because the strong electric field at the interface may increase the polaron mobility, and thus also may influence the barrier height of the band discontinuity at the interface. Since a finite current density in the reverse direction can only be due to electron–hole polaron pair generation at the interface, we interpret the rate *r* as being limited by the generation rate of polaron pairs, which are separated in the SCR. The voltage dependence of the polaron pair generation as well as the bias dependent drop of *E*_B_ can both give rise to a rate *r* > 1. For PCMO, exciton binding energies can be neglected because of the high dielectric constant of ε = 30 [63].

By lowering the temperature the rates increase until a temperature of 80 K is reached. For 80 K and 60 K, a constant rate is visible. At a temperature of 40 K, the rate drops by several orders of magnitudes. The constant value of *r* at a low temperature and the reduction below 60 K can indicate the transition from thermally induced separation at the interface to tunneling through the barrier. This is supported by the large *n* values. We assume that the determined barrier height, *E*_B_ = 283.5 meV (see Figure 11c), in the reverse direction is strongly decreased due to the buildup of a large electric field at the junction interface.

Previous studies of a PCMO–STNO junction show the rectifying character of the *J*–*V* curve can predict a p–n diode-type band diagram for this type of junction [64]. In contrast to our study, a lower doping level of Nb (*y* = 0.0002) was used, which leads to a more extended space charge region in the STNO. The rectifying *J*–*V* characteristic shows no noticeable breakdown in the reverse direction up to very high voltages and an ideality factor of *n* = 1.05–1.10 indicates thermally induced separation processes across the junction.

In our study, a clear contribution of the series resistance in the forward direction at higher voltages is attributed to the bulk resistance of the PCMO thin film. Furthermore, the higher Nb doping reduces the resistance contribution of the STNO bulk. In addition, the space charge region in the STNO is much smaller and therefore the probability for tunneling processes across the interface at lower temperatures is increased. Both effects are an important prerequisite for the analysis of the polaronic carriers on the energy conversion in a correlated oxide solar cell.

### Evaluation of the measured diffusion length

The sum of the diffusion lengths in PCMO and STNO *L*_PCMO_ + *L*_STNO_ = 21 nm measured by EBIC is closer to the values found in polymer–fullerene solar cells than the ones typical for inorganic semiconductors. In our simulation, we neglect any surface recombination processes, which could reduce the exponential decay width in the measured EBIC linescan, leading to apparently smaller diffusion lengths. Consequently, we interpret the result found here as a lower limit to the real diffusion lengths.

The asymmetry in the measured EBIC signal suggests *L*_PCMO_ is smaller than *L*_STNO_, coinciding with much smaller charge carrier mobility in PCMO than in STNO. The small diffusion length substantiates the importance of the polaron carrier model for this material class. Furthermore, it suggests that a substantial part of the current stems from excitations in the SCR. This emphasizes the importance of conduction processes in or near the SCR in manganite–titanate junctions.

## Conclusion

In this contribution we have analyzed the current–voltage characteristics of a PCMO–STNO junction in the framework of the one diode model based on Shockley theory. The model seems to be applicable for these types of junctions given the consistency of the determined parameters and their reasonable temperature behavior. However, modifications must be taken into account. The microscopic interpretation of each of these parameters must include the strong correlation effects of the involved materials and their local change at the interface. Our EBIC measurements show a relatively small diffusion length comparable to that of organic semiconductors with low mobilities. The series resistance, *R*_S_, reflects the thermally activated hopping mobility of small polarons in PCMO. In addition, the CER-like behavior of the parallel resistance, *R*_P_, also points to strong correlation effects. In addition, the absence of the CER-like behavior in *R*_S_ is an indication that the main contribution of *R*_P_ originates from the interface. The very strong voltage dependence of the reverse saturation current can be explained by the generation and separation of electron and hole polaron pairs at the interface with a voltage-dependent generation rate, *r*. The dominate contribution to the current in the reverse direction is the generation current in the SCR of PCMO and this current is both limited by the electron–hole polaron pair generation and their separation due to the interfacial energy spike of the conduction band. More work must be performed in the future in order to develop a microscopic understanding of the origin of the interfacial energy spike, which may involve the effects of band offset, orbital mismatch between states of different symmetry as well as local correlation effects.

## Experimental

An epitaxial thin film of p-doped Pr_1−_*_x_*Ca*_x_*MnO_3_ (PCMO, *x* = 0.34) was deposited on single crystalline n-doped SrTi_1−_*_y_*Nb*_y_*O_3_ (STNO, *y* = 0.002) to fabricate p–n heterojunctions. The thin film was deposited via reactive ion beam sputtering from a stoichiometric target with a film thickness of *t* ≈ 100 nm. During the deposition, the process temperatures was *T*_dep_ = 750 °C, the pressure of the Xe sputtering gas was *p*_Xe_ = 1 × 10^−4^ mbar and the pressure of the oxygen background gas was *p*_O2_ = 1.4 × 10^−4^ mbar. Ohmic contacts were provided by sputtered Ti contacts with a Au protection coating on the STNO substrate and Au contacts on the PCMO. Here the area of the contacts was 1 × 4 mm^2^ and they were structured by the use of a shadow mask at a process temperature of *T*_dep_ = 200 °C. After deposition, the quality of the films was checked by XRD techniques.

**Figure 12:**
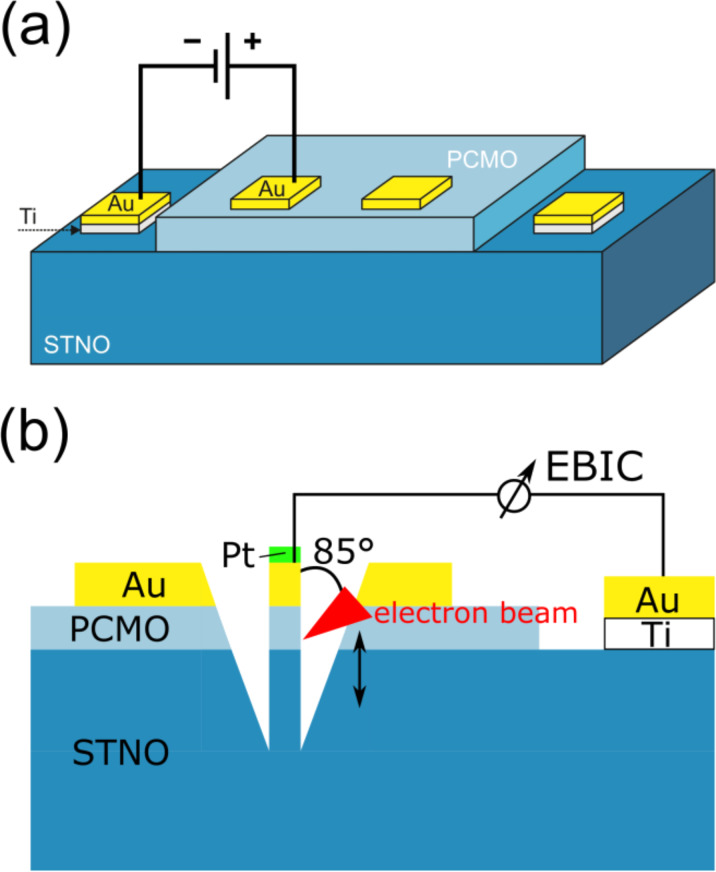
(a) Sketch of the sample geometry for the electrical measurements in the cryostat, and (b) sketch of the sample geometry for the cross-section EBIC measurement. The lamella thickness along the beam direction was estimated from SEM measurements to be about 330 nm at the PCMO–STNO interface.

For electrical characterization, the sample was connected in a two point geometry, where the resistances of the supply cables were excluded in a cryostat with a Suprasil entry window. The geometry is shown in Figure 12a. During the measurement, the maximum current density flow through the junction was set to *J* = 2500 A/m² and the voltage range was between *V* = ±1 V. For every measurement, the positive pole was connected to the contact on the PCMO. To illuminate the sample, a Xe arc lamp with a power density of *p*_ph_ ≈ 155 mW/cm² was used.

The EBIC measurements were carried out in a FEI Nova Nanolab 600 dual beam focused ion beam microscope. Standard EBIC equipment from Gatan was used, including a Stanford SR570 current amplifier. The cross section (see Figure 12b for a sketch of the geometry) was prepared using a standard focused ion beam TEM lamella preparation at 30 kV ion acceleration voltage, with the final cleaning of the surfaces at 5 kV.
